# Measurement of lateral acetabular coverage: a comparison between CT and plain radiography

**DOI:** 10.1093/jhps/hnv063

**Published:** 2015-10-22

**Authors:** Vivek Chadayammuri, Tigran Garabekyan, Mary-Kristen Jesse, Cecilia Pascual-Garrido, Colin Strickland, Kenneth Milligan, Omer Mei-Dan

**Affiliations:** 1. Department of Orthopaedic Surgery, University of Colorado School of Medicine, Aurora, CO, USA; 2. Department of Orthopaedic Surgery, Division of Sports Medicine and Hip Preservation; 3. Department of Radiology, Division of Musculoskeletal Radiology, University of Colorado School of Medicine, Aurora, CO, USA

## Abstract

We prospectively evaluated the degree of absolute agreement between measurements of lateral center-edge angle (LCEA) on plain radiography (XR) and computed tomography (CT) in a consecutive cohort of 205 patients (410 hips) undergoing hip arthroscopy. Preoperative measurements of the LCEA were performed bilaterally utilizing standardized anteroposterior radiographs and coronal reformatted CT scans. Demographic variables including age, gender, height, weight, BMI and clinical diagnosis were recorded for all patients. Overall, measured values of the LCEA were 2.1° larger on CT compared with XR (32.9° versus 30.8°, *P* < 0.001). Subgroup analysis revealed the highest mean difference in hips with acetabular dysplasia and concomitant cam-type femoroacetabular impingement (FAI) [mean difference (CT–XR) 5.5°, 95% confidence interval (CI) 3.7°–7.3°, *P* = 0.011], followed by hips with isolated acetabular dysplasia (mean difference [CT–XR] 4.9°, 95% CI 2.7°–7.0°, *P* < 0.001). In contrast, 119 (29.0%) of the hips demonstrated larger measurements of the LCEA on 25 XR relative to CT. Of these hips, 20 (16.8%) had pincer-FAI and 25 had cam-FAI (21.0%), representing a significantly higher proportion compared with all other clinical subgroups (*P* = 0.045 and 0.036, respectively). Our study demonstrates measured values of the LCEA are consistently inflated on CT relative to XR for a wide variety of hip pathologies, highlighting the need for standardization and validation of CT-based measurements to improve the quality of clinical decision making.

**Level of Evidence:** Diagnostic Level II.

## INTRODUCTION

The lateral center-edge angle (LCEA) of Wiberg is a widely utilized measurement for the evaluation of acetabular coverage [1–3]. First described in 1939, the LCEA is defined as the angle subtended by a vertical line passing through the center of femoral head and a line extending from the center of the femoral head to the lateral acetabular rim [1, 3, 4]. Measurement of the LCEA >40° is considered indicative of pincer-type femoroacetabular impingement (FAI) and may be associated with protrusio acetabuli or global acetabular overcoverage [2, 5], while measurement of the LCEA <20° is confirmative of acetabular dysplasia.

 Although measurement of the LCEA was initially established and validated using standard anteroposterior pelvic (AP) radiography, recent trends have favored its measurement on computed tomography (CT) [6–8]. The increasing popularity of CT is, in part, attributed to its improved accuracy for visualization of bony morphology and preoperative planning relative to other imaging modalities in patients undergoing hip surgery [7, 9–11]. In spite of this, it remains unclear whether measured values of the LCEA on CT are concordant with those obtained on plain radiography.

The purpose of this study was to evaluate whether a significant difference exists between measurements of the LCEA on plain radiography and CT in a large cohort of patients undergoing hip arthroscopy. The null hypothesis was that measurement of the LCEA on CT and plain radiography would be statistically equivalent, irrespective of clinical etiology.

## METHODS

After Institutional Review Board approval was obtained, we performed a prospective single-center observational cohort study on a consecutive series of 205 patients (410 hips) presenting to our dedicated hip preservation service. Inclusion criteria for patients selected for this study were as follows: (i) persistent hip pain and mechanical symptoms refractory to non-operative management lasting at least 3 months, (ii) reproducible clinical examination findings suggestive of impingement or instability, (iii) joint-space width exceeding 2 mm on all views of plain radiography and three-dimensional (3D) CT and (iv) availability of preoperative measurements of the LCEA on plain radiography and CT. Patients presenting with severe anatomic deformity such as slipped capital femoral epiphysis (SCFE), Legg–Calves–Perthe’s disease, osteochondromatosis or post-dislocation syndrome were excluded from this study.

### Imaging protocol

After a comprehensive history and physical examination were performed, patients underwent a standardized series of plain radiographs (including supine AP and cross-table lateral views) and preoperative CT scans with 3D surface-rendered reconstruction of the entire pelvis, proximal femurs and knees. The standard AP pelvic view was obtained with the patient positioned supine with the lower extremities internally rotated 15° to maximize femoral neck length. The X-ray beam was directed midway between the anterior superior iliac spine and the pubic symphysis, with a focus film distance of 100 cm. Films were considered adequate given symmetric obturator foramina and a distance of 1.0–3.0 cm between the coccyx and pubic symphysis [3, 12]. For CT acquisitions, patients were placed on the CT gantry in supine position with care taken to assure a square pelvis relative to the table. The feet were secured in neutral (toes up) position in a plastic foot binder. Whole pelvis 1-mm acquisitions with 2-mm reconstructions in axial, sagittal and coronal orthogonal planes were obtained, and additional oblique axial 2-mm reconstructions were performed along the long axis of both femoral necks [10]. Single series 3-mm cuts were acquired through the patient’s knees (field of view from 2 cm above the patellar apex to 2 cm below the fibular head) for the purpose of calculating the femoral torsion angle. Finally, 3D surface-rendered reconstructions of the entire pelvis were performed to allow detailed morphologic evaluation and to aid in surgical planning. On all imaging modalities, the femoral head center was approximated using Mose templates [13].

Joint space width was defined as the narrowest distance between the bony contour of the acetabular rim and femoral head at the weight-bearing zone. The LCEA was determined on AP pelvic radiography according to the modification described by Ogata *et al.* [14], given by the angle subtended by (i) a line drawn through the center of the femoral head and orthogonal to the transverse line passing through the teardrops of both hips and (ii) a line drawn from the center of the femoral head to the lateral weight-bearing sclerotic zone (sourcil) of the acetabular rim. The LCEA on CT was measured on a single reformatted coronal image through the center of the femoral head, a location determined by a corresponding scout line transecting the greatest diameter of the femoral head on axial reference images, and orthogonal to the standard axial plane. The LCEA was determined as the angle measure between a line perpendicular to the transverse axis of the pelvis and a line extending from the center of the femoral head to the lateral margin of the acetabular roof. All measurements were determined using the digital caliper Philips PACS system (Philips iSite PACS; Philips Healthcare, Andover, MA, USA).

### Patient diagnosis

Clinical diagnoses of bony impingement and/or acetabular dysplasia were determined according to accepted pathomorphologic signs and measurements [2, 4]. Suggestive physical examination findings included reductions in hip flexion and internal rotation range of motion and/or positive impingement and other provocative tests [15]. Confirmative imaging findings of pincer anatomy included acetabular retroversion (crossover sign or ischial spine sign), LCEA exceeding 40° and/or acetabular inclination <0°; features of cam-FAI included an alpha angle exceeding 50° on radial sequences of the head–neck junction and a femoral head–neck offset ratio <0.18; and features of lateral acetabular dysplasia included an LCEA <20°. Common indications for hip arthroscopy included in this study were FAI, hip instability due to dysplasia (prior to periacetabular osteotomy) and/or excessive femoral torsion (prior to derotational femoral osteotomy).

The diagnosis of symptomatic hip instability due to dysplasia was established by a clinical history of pain, positive findings on provocative hip tests indicating labral tear, measurement of LCEA <20° on AP pelvic radiography and magnetic resonance imaging (MRI) findings of labral hypertrophy, articular cartilage thickening or partial ligamentum teres tear. Patients with the above findings who also demonstrated evidence of an engaging cam lesion on intraoperative impingement testing were further classified as cam-type FAI with concomitant hip dysplasia.

The degree of acetabular coverage was determined by LCEA measurement: normal acetabular coverage (25°–40°), acetabular overcoverage (≥40°) borderline dysplasia (20°–24.9°) and frank dysplasia (<20°). The history, physical examination, imaging and intraoperative findings (for hips treated surgically) were all important considerations in arriving at the final diagnosis for each hip according to the following categories: no pathology, mixed-FAI, cam-FAI, pincer-FAI, hip dysplasia (borderline or frank) and FAI with concomitant hip dysplasia (borderline or frank) (Table I).

**Table I.** Patient demographics and baseline characteristics

Patient variables	Data
Total number of patients (no. of hips), *N*	205 (410)
Male gender, n (%)	62 (30.2)
Age, mean (SD), years	32.4 (10.6)
Height, mean (SD), cm	169.9 (9.8)
Weight, mean (SD), kg	70.7 (15.8)
BMI, mean (SD), kg/m^2^	24.3 (4.5)
Symptom onset, median (IQR), months	18.0 (29.0)
Clinical diagnosis, no. of hips (%)	
No pathology	114 (27.8)
FAI	265 (64.6)
Mixed	138 (33.6)
Cam	75 (18.3)
Pincer	52 (12.7)
Hip dysplasia	18 (4.4)
FAI with concomitant hip dysplasia	13 (3.1)

### Examiners

Clinical examination and radiographic findings were determined by a senior hip preservation orthopedic surgeon. CT measurements were evaluated by our institution’s dedicated musculoskeletal radiology team composed of four fellowship-trained musculoskeletal radiologists with 6–12 years of experience and were verified in complex cases by two senior authors. Assessors of radiographs and CT scans were blinded to each other’s measurements.

Interobserver reproducibility of LCEA measurement on plain radiography and CT was evaluated by the two senior authors in a blinded random subset of 25 hips using a two-way, mixed, consistency single-measures intraclass correlation coefficient (ICC). ICC values greater than 0.80 indicate excellent reliability, 0.61–0.80 substantial reliability, 0.41–0.60 moderate reliability, 0.21–0.40 fair reliability and <0.20 poor reliability [16]. Accordingly, the ICC demonstrated excellent reliability for measurements of the LCEA performed on CT [ICC = 0.992, 95% confidence interval (CI) 0.981–0.996] and plain radiography (ICC = 0.934, 95% CI 0.850–0.971).

The following demographic data were recorded for all patients: age, clinical diagnosis, side of surgery, gender, height, weight and body mass index (BMI).

### Statistical analysis

All variables were evaluated for distribution of normality using a combination of histograms, quantile–quantile (Q–Q) plots and the Shapiro–Wilk test (with normality given by *P* > 0.05). Descriptive statistics for continuous and categorical variables were summarized as means and standard deviations or counts and frequencies, respectively. The mean difference between measurements of the LCEA on CT and plain radiography was visually inspected using Bland–Altman plots and analyzed using the Student’s paired *t*-test. The proportion of hips with higher measurements of the LCEA on plain radiography compared with CT was compared using the chi-square test or Fisher’s exact test (expected cell count < 5). Statistical significance was set at *P* < 0.05 (two tailed). All statistical analyses were conducted using IBM SPSS Statistics (Version 22.0, IBM, Inc.).

## RESULTS

### Participants and descriptive data

The study cohort comprised 205 patients (62 men, 143 women) with a mean age of 32.4 years (range 11–61). The mean patient height was 169.7 cm (range 149.9–195.6), mean patient weight was 70.4 kg (range 40.8–118.4) and mean patient BMI was 24.3 kg/m^2^ (range 16.4–44.8). Among all 410 hips, 114 (27.8%) were asymptomatic, 138 (33.6%) had mixed-FAI, 75 (18.3%) had cam-FAI, 52 (12.7%) had pincer-FAI, 18 (4.4%) had isolated acetabular dysplasia and 13 (3.1%) had acetabular dysplasia with concomitant FAI. Additional baseline characteristics are summarized in Table I.

Overall, measured values of the LCEA on CT were 2.1° larger than the corresponding values on plain radiographs (32.9° versus 30.8°, *P* < 0.001; Table II). When analyzed according to clinical etiology, a majority of symptomatic hips demonstrated a larger measurement of the LCEA on CT relative to plain radiographs, with the highest mean difference of 5.5° (95% CI 3.7°–7.3°, *P* = 0.011) occurring in hips with acetabular dysplasia and concomitant FAI and the second highest difference of 4.9° (95% CI 2.7°–7.0°, *P* < 0.001) occurring in hips with isolated acetabular dysplasia. Asymptomatic hips also showed a significant increase in measurement of the LCEA favoring CT compared with plain radiography (mean difference 2.4°, 95% CI 1.6°–3.1°, *P* < 0.001). In contrast, the difference between measurements of the LCEA on plain radiographs and CT did not vary significantly in hips with pincer-FAI (mean difference 0.7°, 95% CI −0.4° to 1.9°, *P* = 0.213) or cam-FAI (mean difference 0.6°, 95% CI −0.2° to 1.4°, *P* = 0.116; Fig. 4A and B).

**Table II.** Mean difference between measurements of LCEA obtained on plain radiography versus CT

	LCEA on plain radiography, mean (SD)	LCEA on CT, mean (SD)	Mean difference (95% CI)	*P*-value
Entire cohort	30.8 (7.1)	32.9 (7.5)	2.1 (1.7–2.5)	<0.001*
Subgroup analysis according to clinical etiology				
No pathology (control)	32.4 (6.4)	34.8 (7.3)	2.4 (1.6–3.1)	<0.001*
FAI (mixed)	34.0 (6.0)	32.2 (6.8)	1.8 (1.1–2.6)	<0.001*
FAI (cam)	27.7 (3.7)	28.4 (3.9)	0.6 (−0.2 to 1.4)	0.116
FAI (pincer)	36.3 (6.4)	37.0 (7.8)	0.7 (−0.4 to 1.9)	0.213
Hip dysplasia	17.5 (1.9)	22.6 (4.0)	4.9 (2.7–7.0)	<0.001*
Cam-type FAI with concomitant hip dysplasia	17.3 (4.0)	22.8 (3.2)	5.5 (3.7–7.3)	0.011*

*Statistically significant, *P* < 0.05 (presented in bold).

**Fig. 1.**
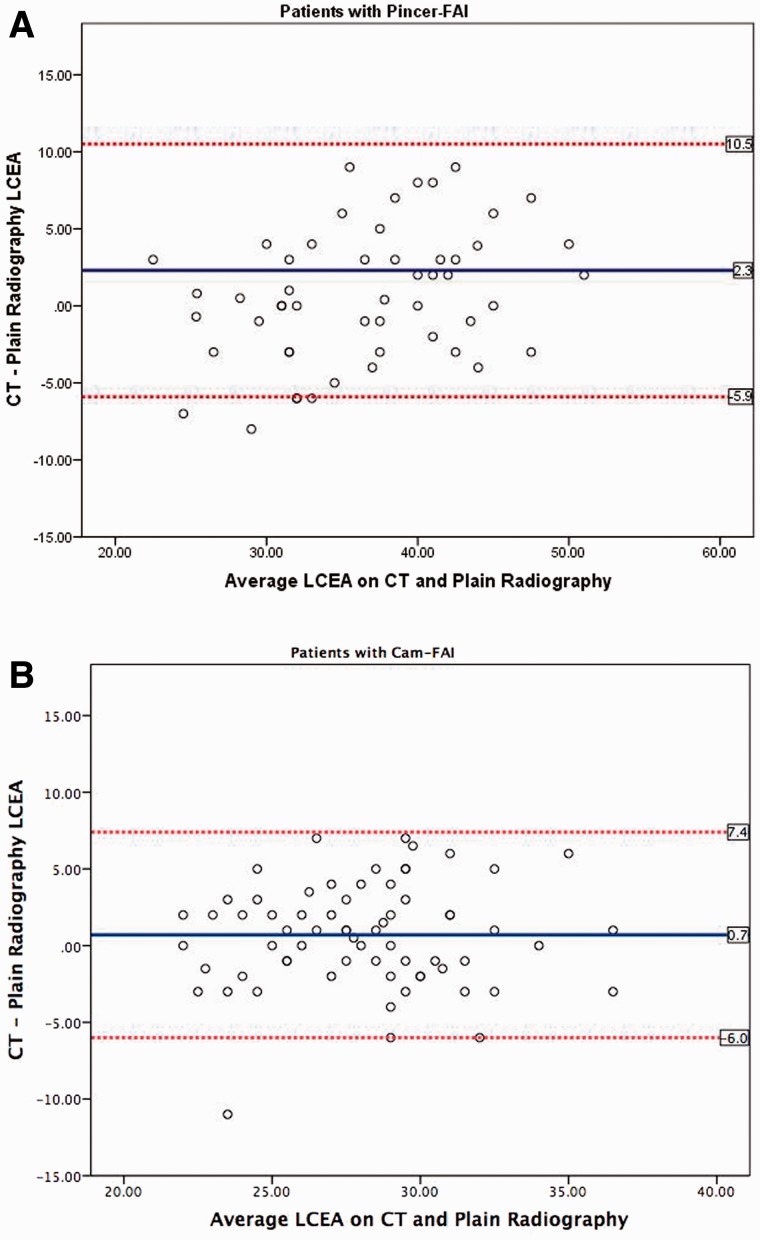
Bland–Altman plots depicting the variability of measurements of LCEA on plain radiography versus CT in patients with (**A**) pincer-FAI and (**B**) cam-FAI. The mean difference between measurements of LCEA on CT and plain radiography (blue line) is nearly 0; however, plotting of the data reveals a relatively even spread of measurements that were greater on plain radiography compared with CT. This is in contrast to the trend observed for all other clinical subgroups, in which measurement of LCEA was uniformly inflated on CT relative to plain radiography.

A subset of 119 (29.0%) hips demonstrated larger measurements of the LCEA on plain radiographs relative to CT. Among them, 20 hips (16.8%) had pincer-FAI and 25 hips (21.0%) had cam-FAI, a significantly higher proportion than all other clinical subgroups (*P* = 0.045 and *P* = 0.036, respectively; Fig. 1A and B). In contrast, no hips categorized as FAI with concomitant acetabular dysplasia, and only two (6.5%) hips with isolated dysplasia, demonstrated increased measurements of the LCEA on plain radiographs compared with CT.

## DISCUSSION

**Fig. 2.**
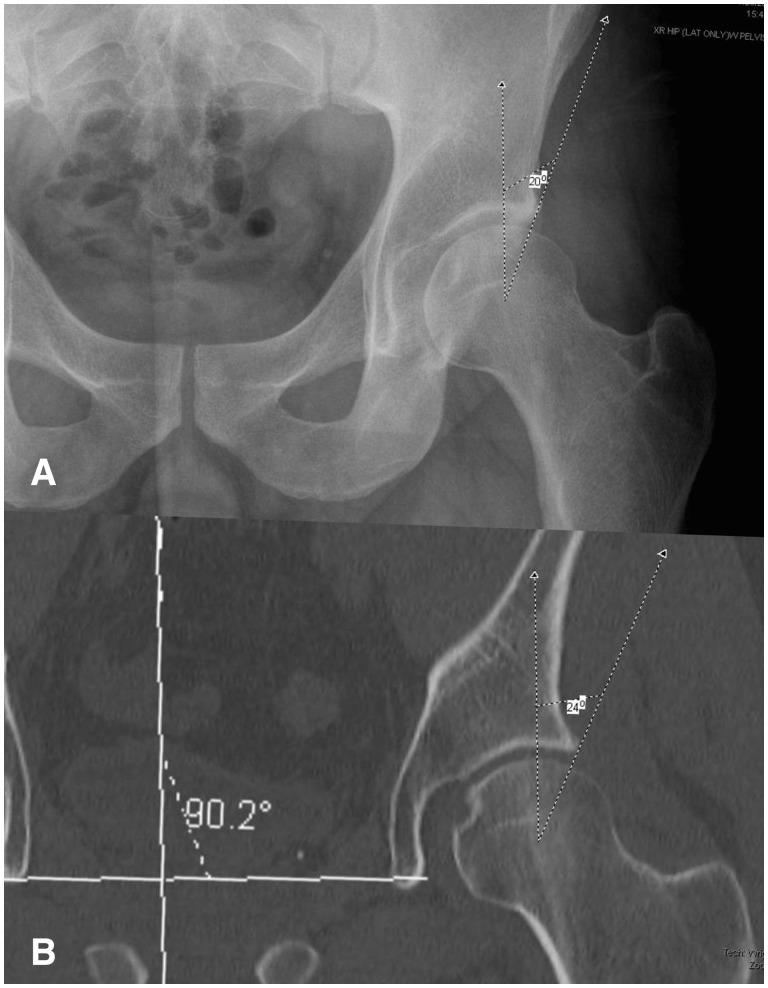
Representative example of lateral acetabular dysplasia. The measured LCEA is (A) 20° on AP pelvic radiography, compared with (**B**) 24° on coronal CT. These measurements confer a clinical diagnosis of frank hip dysplasia and borderline hip dysplasia, respectively—a discrepancy that could alter the course of operative treatment.

**Fig. 3.**
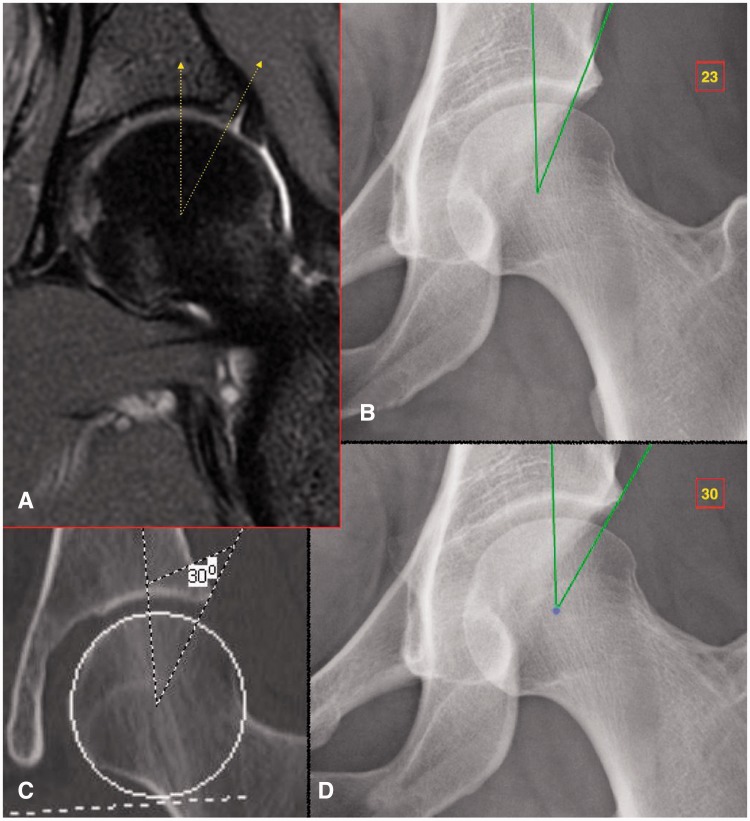
Representative example in borderline dysplastic hip. (**A**) Coronal MRI of the left hip shows the attachment point of the labrum to the lateral margin of the acetabular roof. (**B**) Measurement of the LCEA (of Ogata) yields a value of 23° when using a terminal endpoint at the weight-bearing area of the acetabulum that, as seen by the MRI, is located at the medial base of the labrum. (**C**) Measurement of the LCEA (of Wiberg) on coronal CT involves a terminal endpoint located at the far lateral acetabular rim, yielding a value of 30°. (**D**) Measurement of the LCEA on plain radiography using a technique analogous to that performed on coronal CT. The difference between LCEA measurements in panels B and D is attributable to a bony area which functions as the labral base but does not come into contact with the femoral head and thus does not contribute directly to the acetabular coverage.

Conventional clinical practice has often assumed an interchangeability between measurements of lateral acetabular coverage on plain radiography and two-dimensional (2D) CT. However, the results of this study demonstrate that measurement of the LCEA varies significantly between the two imaging modalities according to clinical etiology. Most notably, the LCEA was larger by a mean of 5.1° on CT compared with plain radiography for hips with acetabular dysplasia and concomitant cam-FAI and 4.9° for hips with isolated acetabular dysplasia. Due to this measurement discrepancy, a majority of hips in our presented cohort that were identified as frankly dysplastic (LCEA <20°) on plain radiography qualified as normal or borderline dysplastic (LCEA >25° and between 20° and 25°, respectively) on CT (Figs. 2 and 3). In such cases, measurement of the LCEA on one imaging modality as opposed to the other would yield a distinctly different treatment strategy.

**Fig. 4.**
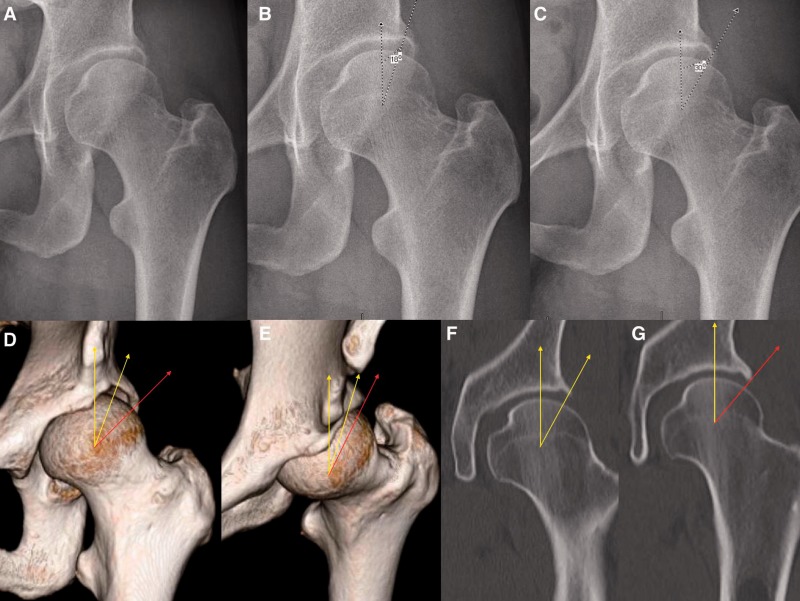
Potential challenges associated with measurement of LCEA in dysplastic hips. (**A**) AP pelvic radiograph of the left hip; (**B**) measurement of the LCEA with a terminal endpoint at the intersection of acetabular rim and anterior acetabular wall yields a value of 18°, indicating acetabular dysplasia, (**C**) while measurement with a terminal endpoint at (what seems to be) the lateral rim yields a value of 30°; (**D**, **E**) 3D CT enables superior visualization of bony morphology. Note that the lateral border of the anterior inferior iliac spine landmark should be positioned at 1 o’clock to ensure measurement of the LCEA to the true anterolateral rim (yellow) at the 12 o’clock position. Failure to do so can potentially result in erroneous measurement of the LCEA with an endpoint on the posterior acetabular wall that does not contribute to anterolateral coverage (red). Corresponding measurement on coronal CT views are shown in panels (**F**) and (**G**).

One potential reason for this discrepancy could derive from the inclusion of the non–weight-bearing lateral acetabular rim in the measurement of lateral acetabular coverage on CT. On coronal CT, the measurement of LCEA is similar to that originally proposed by Wiberg *et al.*—that is, with the weight-bearing zone of the acetabulum assumed to be located at the far lateral margin of the acetabular rim. In contrast, Ogata *et al.* proposed a modified radiographic measurement of the LCEA following the observation that the weight-bearing sclerotic zone of the acetabulum was often better represented by the lateral sourcil, a landmark located medial to the far lateral projection of the acetabular rim. The authors postulated that the use of this modified LCEA measurement could afford improvements in the quality of closed and open reduction of congenitally dislocated hips [14]. In support, Omeroglu *et al.* [17] determined a mean difference of 8.3° between the classic (Wiberg angle) and refined (Ogata angle) measurements of LCEA on AP pelvic radiography in a study of 66 dysplastic hips.

Unfortunately, no standardized protocols currently exist regarding coronal plane alignment or slice selection on 2D CT for measurement of the LCEA. Traditionally, a reformatted coronal image orthogonal to the axial plane and through the center of the femoral head (at its greatest diameter on axial reference images) is utilized, with the underlying assumption that this plane coincides with the lateral acetabular rim and captures true (functional) lateral acetabular coverage. However, this assumption is prone to be invalid in hips with excessive femoral and/or acetabular version abnormality that displaces the center of the femoral head relative to the lateral acetabular rim. Although this relationship has not been previously investigated on CT, a study by Stelzeneder *et al.* [18] demonstrated that slice selection on 2D imaging (MRI) could influence measurement of acetabular coverage. Similarly, patients with significant lumbar lordosis or lumbar kyphosis, and associated pelvic tilt abnormalities, may exhibit apparent overcoverage or undercoverage, respectively [19, 20]. At present, it is unclear how these secondary abnormalities should be accounted for in standardizing CT evaluation of the pelvis. Taken together, these findings illustrate the need to develop a standardized technique for the measurement of the LCEA on CT that subtracts potential variations in 3D hip morphology and pelvic tilt abnormality.

Interestingly, hips with pincer-FAI or cam-FAI demonstrated statistically equivalent measurements of lateral acetabular coverage on plain radiography and CT. Further analysis, however, revealed that these subgroups demonstrated widely variable measurements of LCEA on both imaging modalities that tended to average out in a similar fashion. This phenomenon, too, may be attributable to difficulties associated with measurement technique. In patients with pincer-FAI, acetabular overcoverage frequently obscures the margins of the anterior and posterior walls, rendering it challenging to identify the lateral sourcil during radiographic measurement of the LCEA. Additionally, patients with pincer-FAI commonly demonstrate secondary changes such as os acetabuli and calcified labra, both of which have been previously reported to portend drastic reductions in intra- and inter-observer reliabilities for the measurement of the LCEA [21]. In patients with cam-FAI, femoral head asphericity renders estimation of the femoral head center using the Mose template method challenging and potentially inaccurate. We addressed this by approximating the femoral head contour according to the weight-bearing surface as previously described by Stelzeneder *et al.* [18]. Nonetheless, measurements of the LCEA on CT and plain radiographs in patients with pincer-FAI and cam-FAI remained variable.

Although a comparative analysis of our results remains difficult given the relative paucity of literature examining inter-modality differences for the measurement of lateral acetabular coverage, one study by Kutty *et al.* [22] reported excellent intra- and inter-observer reliability for measurements of the LCEA performed on plain radiographs in a cohort of 55 patients with isolated pincer-FAI. In their study, the mean LCEA preoperatively was 46.2° for hips with pincer-FAI, considerably higher than that determined in this study. Of note, the former patient cohort demonstrated excessive pelvic tilt in several cases, with the distance from the sacrococcygeal joint to the pubic symphysis ranging between 3.2 and 4.7 cm. Although a formal study of pelvic tilt was not undertaken in our study, empirical observation suggested a milder degree of acetabular retroversion and pincer-type deformity in our study cohort. Indeed, increased pelvic tilt has been previously shown to yield higher measurements of the LCEA on plain radiography [23]. A potential solution to this is to evaluate bony morphology and acetabular coverage using 3D CT. In this approach, the 1 o’clock position is defined as being directly in line with the lateral edge of the anterior inferior iliac spine and the LCEA is measured to the anterolateral acetabular rim given by the 12 o’clock position [24–26]. This method may help to circumvent potential misinterpretations associated with 2D imaging, especially in patients with acetabular dysplasia (Fig. 4).

Taken together, our findings suggest that measurements of lateral acetabular coverage may be prone to significant variation and potential inaccuracy owing to obscured margins of the acetabular rim, femoral head asphericity, pelvic tilt variation and secondary morphological changes in the labrum and bony acetabulum. Newer methods for measurement of lateral acetabular coverage, ideally involving the use of 3D CT, are warranted to improve the quality of preoperative imaging and subsequent clinical decision making.

Strengths of this study include its prospective study design, large sample size relative to previously published studies and inclusion of a wide variety of hip pathologies [6, 7]. However, we acknowledge the following limitations. First, our presented cohort is from a single surgeon’s experience at a dedicated hip preservation service and may not be entirely representative of patient populations encountered at other institutions. Indeed, several of our patients were referrals from other hip preservation centers following failed therapy or chronically recurring symptoms. A second notable limitation is that the rotational position of the hips was neutral (toes pointing up) during CT scanning and internally rotated 15° during plain radiography. The internal rotation was necessary to visualize the femoral neck in profile for accurate neck-shaft-angle measurement and is not believed to significantly affect radiographic measurement of the LCEA [27]. Finally, patients with more significant pathologies and deformities such as SCFE and Legg–Perthes–Calves disease were excluded from this study. These clinical subgroups may demonstrate differing trends than those reported herein [7]. Nonetheless, we consider our results to be clinically meaningful and hope that this study prompts further investigation into methods that optimize the measurement of lateral acetabular coverage on CT and plain radiography.

## CONCLUSION

Measured values of the LCEA are consistently inflated on CT relative to plain radiography for a wide variety of hip pathologies. In particular, patients with acetabular dysplasia exhibit a clinically significant discrepancy. Distinct diagnostic criteria must be established to improve the sensitivity of each imaging modality and ultimately guide treatment planning.

## CONFLICT OF INTEREST STATEMENT

One or more authors (O.M.D.) have received funding from Arthrocare and Smith & Nephew, outside the presented work. All other authors report no commercial associations (e.g. consultancies, stock ownership, equity interest, patent/licensing arrangements, etc.) that may pose a conflict of interest in connection with the submitted article. This study was completed under Institutional Review Board approval, Protocol #15-0386.
